# Characterisation of porous knitted titanium for replacement of intervertebral disc nucleus pulposus

**DOI:** 10.1038/s41598-017-16863-8

**Published:** 2017-11-30

**Authors:** Gauri Tendulkar, Vrinda Sreekumar, Frank Rupp, Arun K. Teotia, Kiriaki Athanasopulu, Ralf Kemkemer, Alfred Buck, Alfred Buck, Hans-Peter Kaps, Jürgen Geis-Gerstorfer, Ashok Kumar, Andreas K. Nussler

**Affiliations:** 1 Siegfried Weller Institute for Trauma Research at the BG Trauma Center, Eberhard Karls Universität Tübingen, Schnarrenbergstr.95, Tübingen, Germany; 2 Section Medical Material Science & Technology, University Hospital Tübingen, Ossianderstr. 2-8, Tübingen, Germany; 3 Department of Biological Sciences and Bioengineering, Indian Institute of Technology Kanpur, Kanpur, 208016 UP India; 4 Hochschule Reutlingen, Reutlingen University, Alteburgstraße 150, Reutlingen, Germany; 5 Buck GmbH and Co.KG, Bondorf, Germany

## Abstract

Effective restoration of human intervertebral disc degeneration is challenged by numerous limitations of the currently available spinal fusion and arthroplasty treatment strategies. Consequently, use of artificial biomaterial implant is gaining attention as a potential therapeutic strategy. Our study is aimed at investigating and characterizing a novel knitted titanium (Ti6Al4V) implant for the replacement of nucleus pulposus to treat early stages of chronic intervertebral disc degeneration. Specific knitted geometry of the scaffold with a porosity of 67.67 ± 0.824% was used to overcome tissue integration failures. Furthermore, to improve the wear resistance without impairing original mechanical strength, electro-polishing step was employed. Electro-polishing treatment changed a surface roughness from 15.22 ± 3.28 to 4.35 ± 0.87 µm without affecting its wettability which remained at 81.03 ± 8.5°. Subsequently, cellular responses of human mesenchymal stem cells (SCP1 cell line) and human primary chondrocytes were investigated which showed positive responses in terms of adherence and viability. Surface wettability was further enhanced to super hydrophilic nature by oxygen plasma treatment, which eventually caused substantial increase in the proliferation of SCP1 cells and primary chondrocytes. Our study implies that owing to scaffolds physicochemical and biocompatible properties, it could improve the clinical performance of nucleus pulposus replacement.

## Introduction

Chronic low back pain is the most common ailment among people aged 40–80 years, affecting men and women alike globally. It is a common cause of disability and one of the top ten disorders causing high socio-economic burden with a global prevalence of 22–65%^1^.

Intervertebral disc degeneration has been implicated as a causative factor in chronic lower back pain, since an inability in the disc function is closely linked with degeneration of its components^1^. Till date, numerous therapeutic strategies like spinal fusion, drug treatments, physiotherapy, cell therapy, arthroplasty etc. have been developed aimed at maintaining disc function by preventing further downstream collapse and reduction of inflammatory pain^2,3^. Nevertheless the potential of these interventions in treating the chronic back pain is debated and continues to progress^4,5^. In addition, a considerable motivation of using biomaterial has been advanced, aimed at restoring the function of intervertebral disc. A number of studies have been reported on scaffolds primarily made of stainless steel, cobalt-chromium alloys, titanium based alloys, hydrogels as potential therapeutic alternatives^6,7^ for such injuries. A more substantial approach through nucleus pulposus replacement^5^ will necessitate as a minimal invasive technique, aiming to decrease the post-surgical risk factors. However, currently available nucleus implants have failed due to associated complications such as corrosion, inflammation, hypersensitivity, fibrous encapsulation, low fracture toughness, mismatched bone and implant modulus of elasticity, leading to revision surgery^7,8^. These factors along with patient response (poor compliance with rehabilitation), surgical techniques and implant surface characteristics results in inadequate benefit of this treatment strategy.

Thus, several modifications of these artificial implants are in progress to overcome the diminutive emergences and limitations^9^. However for a scaffold to be used in tissue engineering applications, it is essential to be biocompatible, have sufficient mechanical strength, integrate with biological tissue and should support cell proliferation^10^. Interaction between the implant and the surrounding tissue *in vivo* mainly depends, not only on the bulk properties of the implant material but also on the surface properties such as surface roughness, composition, surface energy, microstructure, porosity and the pore size^11^. Modifications in material surface physical properties persuade various cellular responses, resulting in changes of cell adhesion and proliferation. Various scaffold surface modifications are promising in terms of modulating and improving wear resistance, inducing long term stability in the host system and osseo integration^12^. Previously, Kettler *et al*.^13^ in their study reported that this unique knitted titanium scaffold possessed adequate biomechanical properties and showed low migration tendency, thereby restoring the physiological axial deformability. Further, we have demonstrated that the knitted scaffold enable the cell attachment and promote cell proliferation^14^. Subsequently, in this current study we have shown for the first time the influence of oxygen plasma surface treatment towards cellular response. Earlier studies have proven that, hydrophilic implant surfaces favor the cellular response like cell adhesion and proliferation of various cell types. Further it has also been reported that the surface wettability plays a role in determining events that takes place at cell material interface and modification of surface wettability effectively enhances the biological response^15,16^. Nevertheless, the contact angles usually observed for titanium and its alloy varies between 70–90 °, indicating weak hydrophilicity^15,16^. The main interest of the current study was therefore to hydrophilize the surface of knitted titanium implant for which, this plasma treatment was applied as a favorable approach to increase the wettability, thus augmenting the mesenchymal as well as primary chondrocyte cell growth. Since, introduction of this knitted titanium implant in the clinical setting necessitates further characterisation with respect to host bio-compatibility and bio-functionality, our present study consequently investigated the physio-chemical properties of the scaffold and cellular responses of human bone marrow derived mesenchymal stem cell as well as the primary chondrocytes upon surface modification by oxygen plasma treatment.

## Results

### Physio-chemical characterization of the scaffold

**Figure 1:**
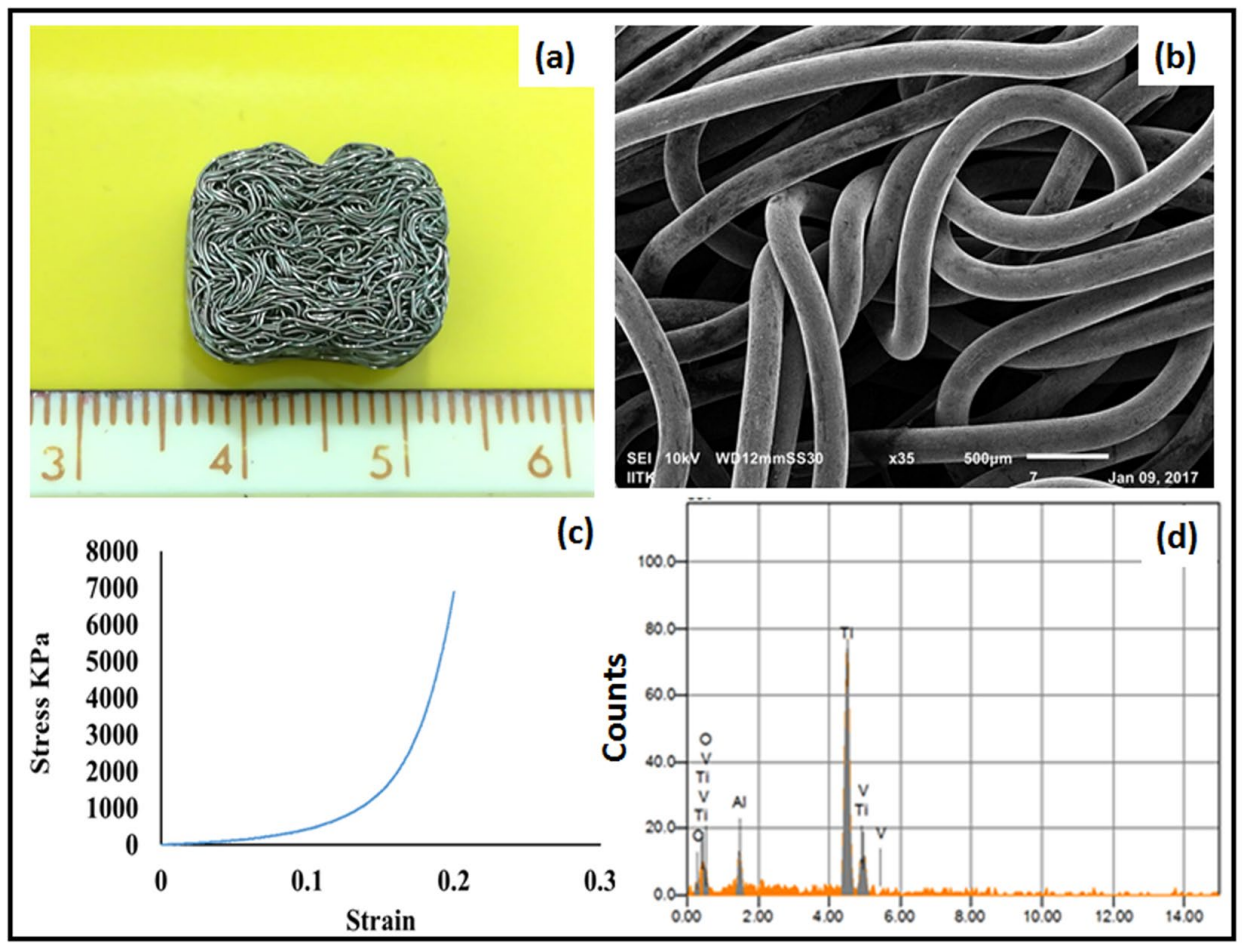
Physical characterization of the titanium scaffold. (**a**) 2D digital image of scaffold structure; (**b**) SEM micrograph depicting knitted scaffold microstructure; (**c**) EDX spectra of the mesh surface; (**d**) Compressive elastic modulus of the scaffold.

**Figure 2:**
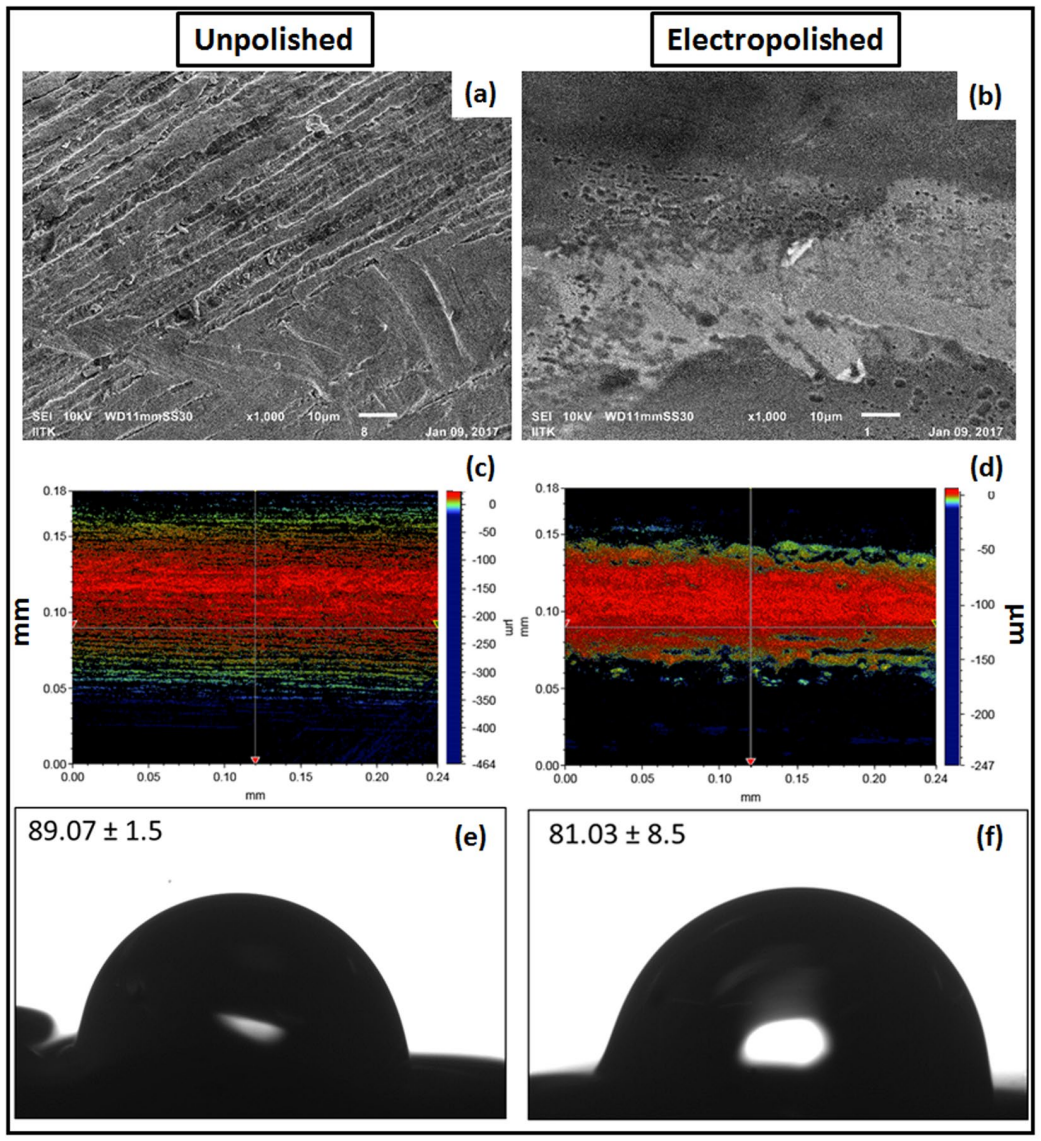
Surface characterization of electro-polished scaffold. (**a**,**b**) SEM image illustrating surface topography; (**c**,**d**) optical profilometry; (**e**,**f**) wettability measurements by sessile drop method; of unpolished and electro-polished scaffold respectively. For optical profilometry, surface area of 240 µm × 180 µm was scanned and the average surface roughness (Ra) was calculated. Ra value of unpolished and electro-polished scaffold was calculated to be 15.22 ± 3.28 µm and 4.35 ± 0.87 µm respectively. Subsequently, wettability measurements showed the weak hydrophilic scaffold surface property which has not affected upon electro-polishing treatment.

Microscopic image of the knitted scaffold revealed the mesh construct and the cross sectional view depicting the non-woven structure. Microstructure analysis using SEM, Fig. 1b illustrates the knitted wire porous architecture of the scaffold having a heterogeneous pore distribution with pore size of 150–400 μm. Surface topography determined at micro scale using SEM and optical profilometry, confirmed that electro-polished scaffold surface is comparatively smooth as that of unpolished scaffold (Fig. 2a,b) and additional, surface finish has been improved without affecting microstructures. The optical profilometry analysis revealed the unpolished surface has average surface roughness (Ra) of 15.22 ± 3.28 µm and the electro-polished surface has average surface roughness of 4.35 ± 0.87 µm (Fig. 2c,d). Energy dispersive X- spectroscopy (EDX) analyses of the unpolished scaffold denoted the elemental constitution mainly consisting of Ti, Al, V (Fig. 1d), which remained unchanged upon electro-polishing treatment. In addition, small traces of oxygen and nitrogen were found as a minor contamination on the scaffold surfaces, which could have originated either during manufacturing process, handling/storage or electro-polishing treatment. Surface energy and wettability are considered as the key factors playing role in cell behavior, cell material interaction and the scaffold integrity. Titanium alloy based scaffold represented weak hydrophilic surfaces; exhibiting contact angles of 83.3 ± 12.19° (Fig. 2e,f). The statistical analysis revealed that there was no considerable difference between the unpolished and electro-polished scaffold wettability (Fig. 2e,f). The observed low wettability of knitted titanium scaffold is a result of a low surface energy. Moreover, the change in surface roughness due to electro-polishing treatment didn’t induce any change in wettability.

Mechanical properties of the scaffold play a crucial role in load bearing application. Compression strength of a biomaterial is therefore considered as one of the main factors. The knitted scaffold illustrated an elastic modulus of approximately 0.02 GPa (Fig. 1c), which is comparable to cancellous bone (0.01–3.0 GPa), but is too low in comparison with cortical bone (4.4–28.8 GPa) as revealed by mechanical analysis^17^.

### Wear debris particle characterization

**Figure 3:**
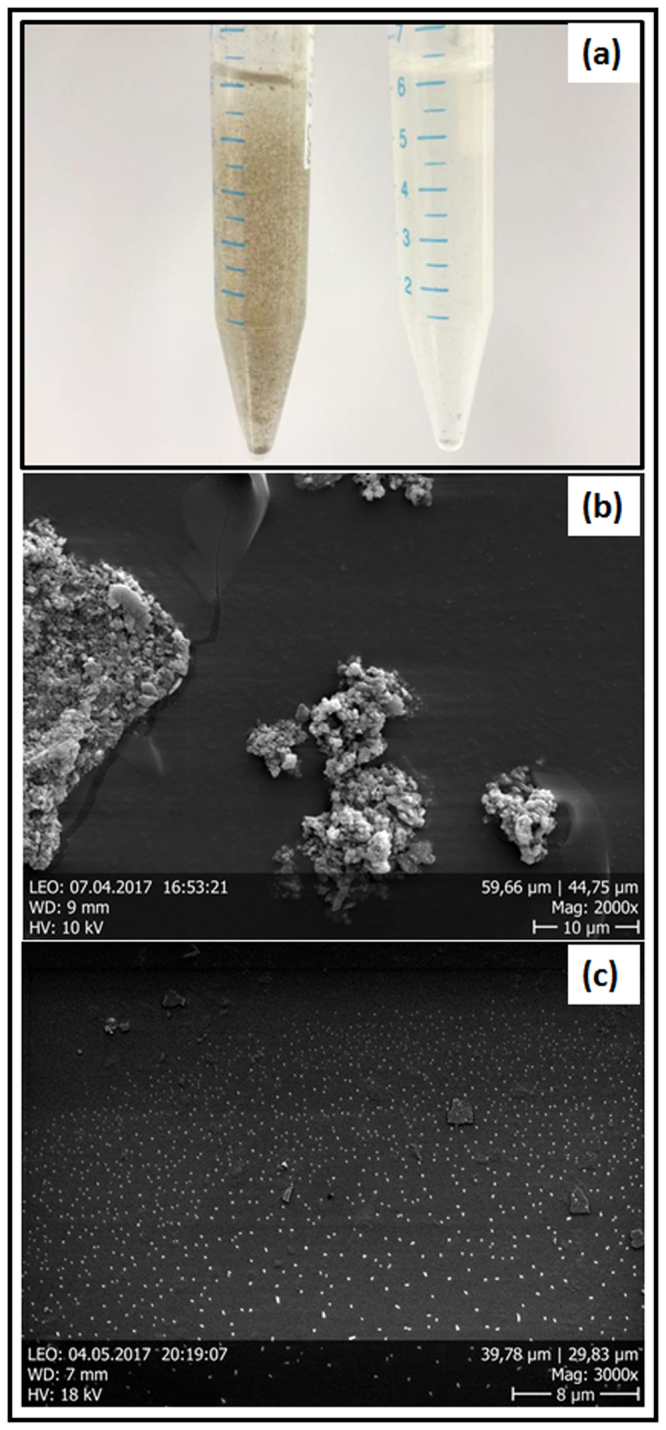
Wear debris particle analysis. (**a**) Digital image of unpolished (left) and electro-polished (right) wear debris particles; (**b**,**c**) SEM images representing morphology and size based distribution of wear debris particles of unpolished and electro-polished scaffold.

The morphologies of the titanium debris showed considerable differences, both in size and shape of the particles. There are two precise differently sized particle groups in the unpolished scaffold based probe (Fig. 3b) ranging from 10–25 µm and sub-micrometer, contrarily there are impartial sub-micron ranged particles in electro-polished scaffold based probe (Fig. 3c). This was already noticeable with the high turbidity of the abrasion solution (Fig. 3a). The particles from unpolished scaffold based probe are very robust and not just clusters of small particles as we initially thought. Since, ultra-sonication as well did not affect these particles thus; we believe that these are solid particles (Fig. 3b). In addition, the particles are far from being spherically shaped. Subsequently, we measured all sort of shape descriptors since the titanium wear particle shape is rather anisotropic.

### Assessment of cell-material interaction

**Figure 4:**
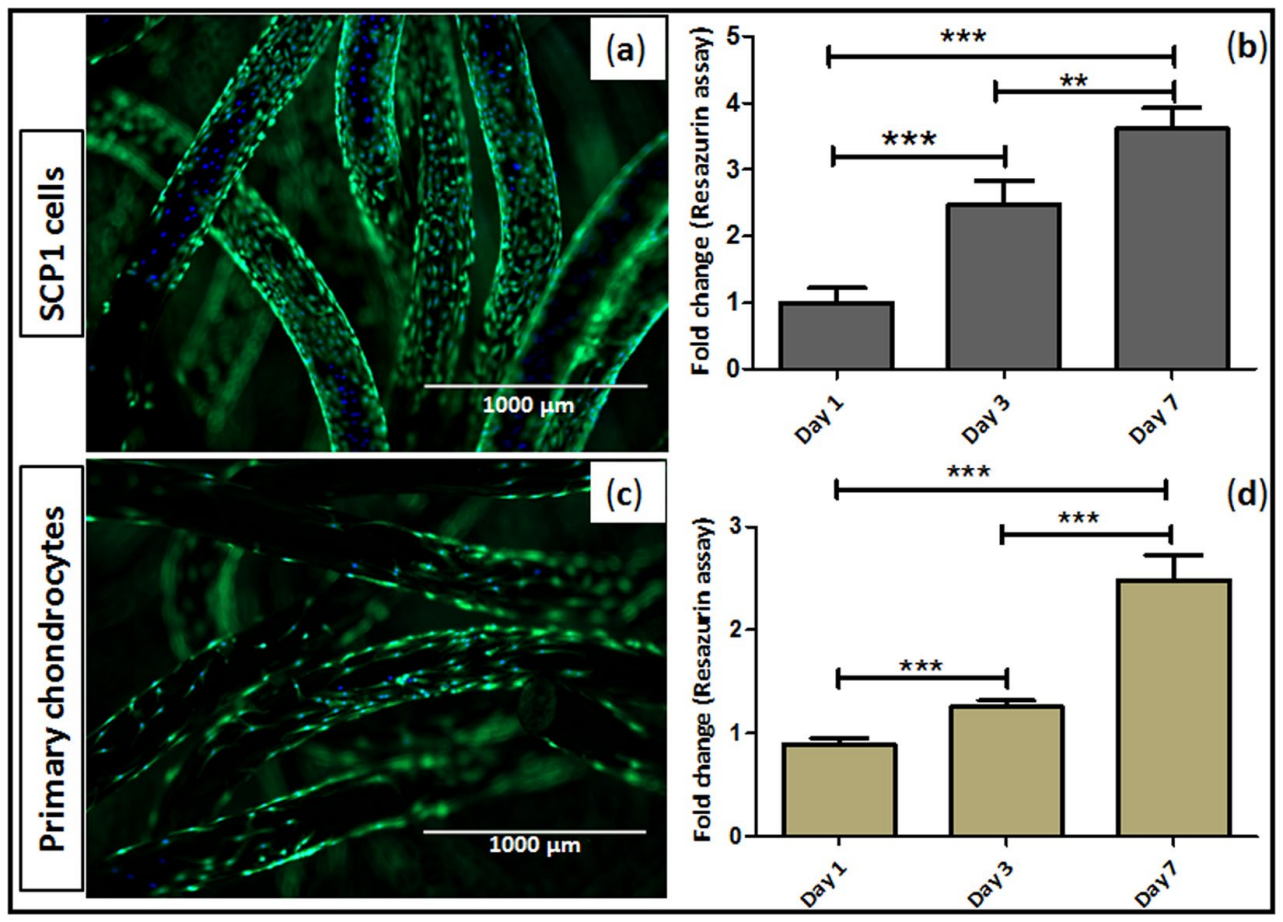
Cell viability and proliferation on the knitted titanium scaffold. (**a**,**c**) Live dead staining - viable cells; calcein-AM (green) and nucleus; Hoechst 33342 (blue) staining of SCP1 cell and human primary chondrocytes upon seeded on the scaffold respectively; (**b**,**d**) Resazurin conversion – indirect assay for cell proliferation of SCP1 cells and human primary chondrocytes respectively. Data represented as mean ± SEM; analyzed by Mann Whitney test. P ≤ 0.05 was considered to be minimum level of significance. *p ≤ 0.05, **p ≤ 0.01, ***p ≤ 0.001. Scale bar = 1000 µm.

The physical characterization of the scaffold was followed by cytocompatibility assessment *in vitro*, where cells were cultured on scaffolds and the cell material interactions like cell adhesion, cell spreading and cell proliferation were observed. The cell culture studies indicated that the cells were able to adhere and survive on the scaffold in spite of low initial cell attachment at day 1. Furthermore, day 3 following cell seeding, we observed a noteworthy increase in the cell growth on the scaffolds. Since electro-polished scaffold exhibited considerably increased wear resistance, we decided to use this scaffold for further cell interaction studies. Cell viability was qualitatively visualized using live-dead assay, where the nucleus was stained blue with Hoechst and cytoplasm stained green with calcein-AM (Fig. 4a,c). Additionally, cell proliferation of human bone marrow derived mesenchymal stem cells as well as of human primary chondrocytes was determined by resazurin assay for cell mitochondrial activity. When the fluorescence was normalized with those on day 1, significant fold change increase in the fluorescence intensity and subsequent cell number was observed, demonstrating the bio-functional potential of the scaffold (Fig. 4b,d).

**Figure 5:**
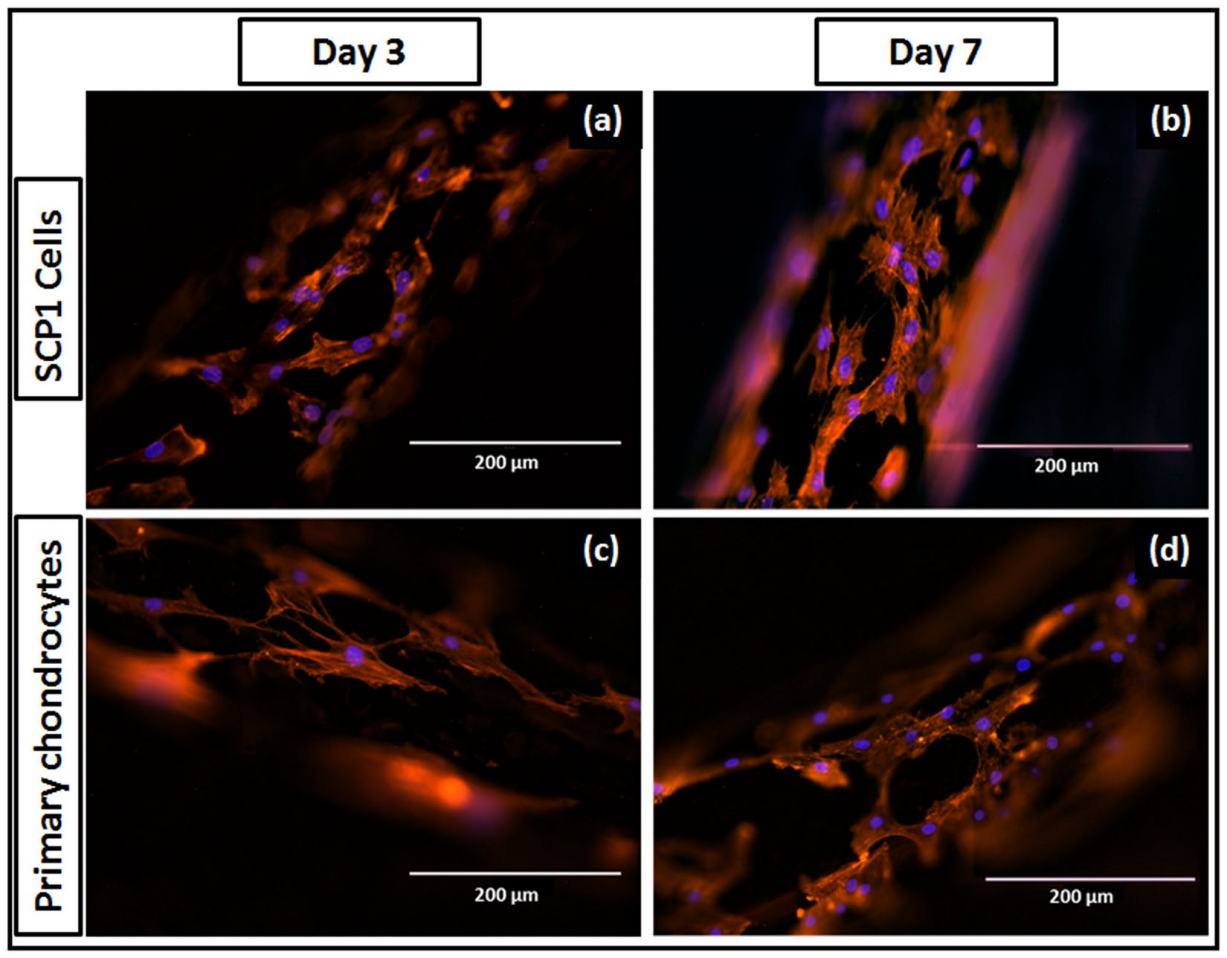
Cell spreading pattern on the knitted titanium scaffold. Image represents actin-phalloidin; alexa 555 (red) and nucleus; Hoechst 33342 (blue) fluorescent staining of (**a**,**b**) SCP1 cells on the scaffold at day 3 and 7 respectively; (**c**,**d**) human primary chondrocytes on the scaffold at day 3 and 7 respectively. Scale bar = 200 µm.

Cell adhesion and spreading on the scaffolds was determined by staining cells with phalloidin actin fibers (red fluorescence) and nucleus (blue fluorescence) at day-3 and day-7 post seeding. It was evident that, cells were effusively spread with extended fliopodia and cytoplasmic protrusions at day 7. In majority cells, orientation was found to be longitudinal along the direction of the knitted wires (Fig. 5). These results further confirm the cytocompatibility of the scaffolds.

### Effect of scaffold surface modification by plasma treatment on the wettability

**Figure 6:**
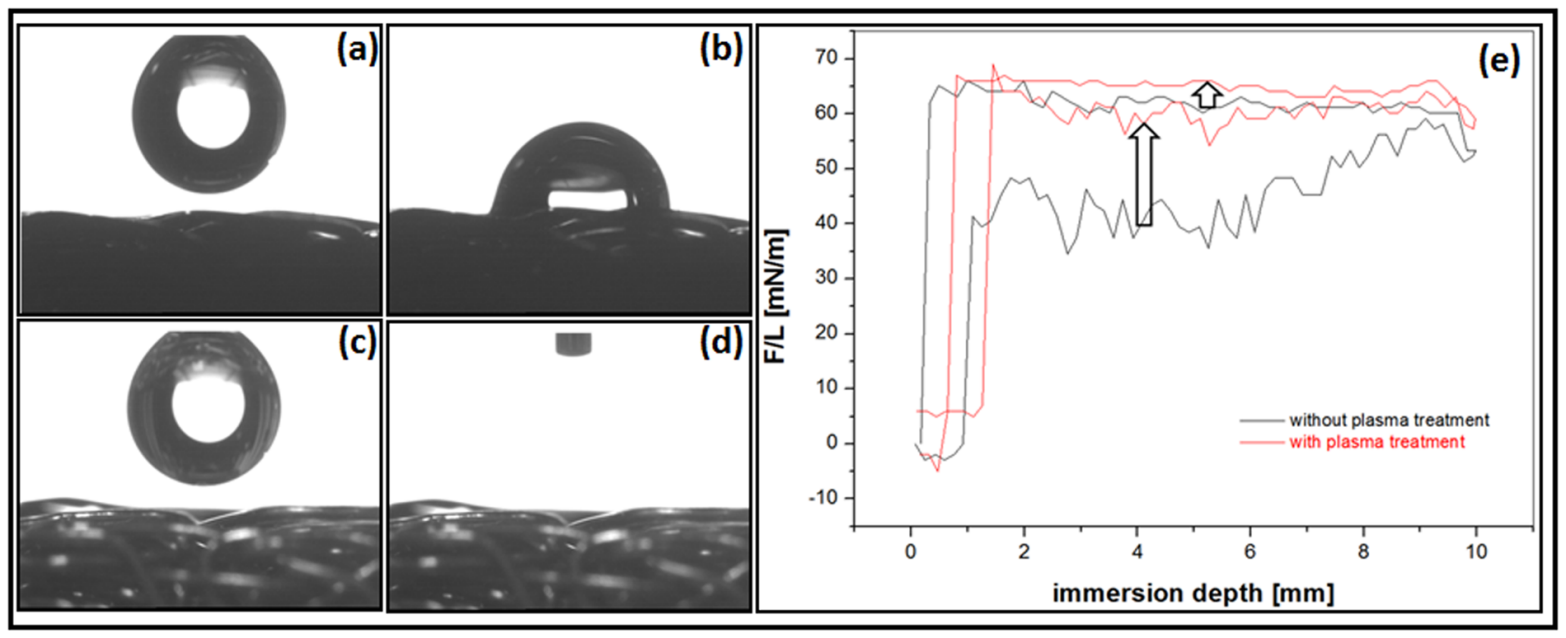
Effect of plasma treatment on the wettability. (**a**) Drop before application on the untreated scaffold by drop shape analysis system; (**b**) the drop after set from the needle onto the untreated scaffold surface; (**c**) drop before application on plasma treated scaffold by drop shape analysis system; (**d**) drop after set onto a plasma treated scaffold was immediately infiltrated into the scaffold; (**e**) tensiometric hysteresis loops of plasma treated and untreated electro-polished wires. The left arrow indicates shifts towards increased wetting tension values F/L of the advancing (adv) and the right arrow of the receding (rec) curves due to plasma treatment.

In order to enhance the cell material interactions, we further modified the electro polished scaffold surface by oxygen plasma treatment. Tensiometric measurements showed shifts to increased wetting tension values (force/length) likewise during immersion (wetting) as well as to a minor degree during emersion (de-wetting) after the plasma treatment (Fig. 6e). As a result of these shifts, hysteresis of the force loops was diminished. Advancing as well as receding contact angles were lowered in consequence to plasma treatments, indicating an overall change towards hydrophilicity. Mean (±standard deviation) advancing contact angles decreased from 72 ± 14° to 52 ± 14°, receding angles from 34 ± 4° to 25 ± 8°.

Whereas the Wilhelmy experiments yielded contact angle data of the pure material surfaces, the sessile drop experiments characterized the wetting behavior of a heterogenic system, *i.e*.; the material/air composite surfaces. Static drops set stable on untreated scaffold surfaces showing contact angles of 83.3 ± 12.19 ° (Fig. 6, see also Fig. 2). In contrast, the experiments clearly indicated water drops being spontaneously infiltrated into the plasma treated scaffold (Fig. 6d).

### Effect of plasma treatment on the cellular response

**Figure 7:**
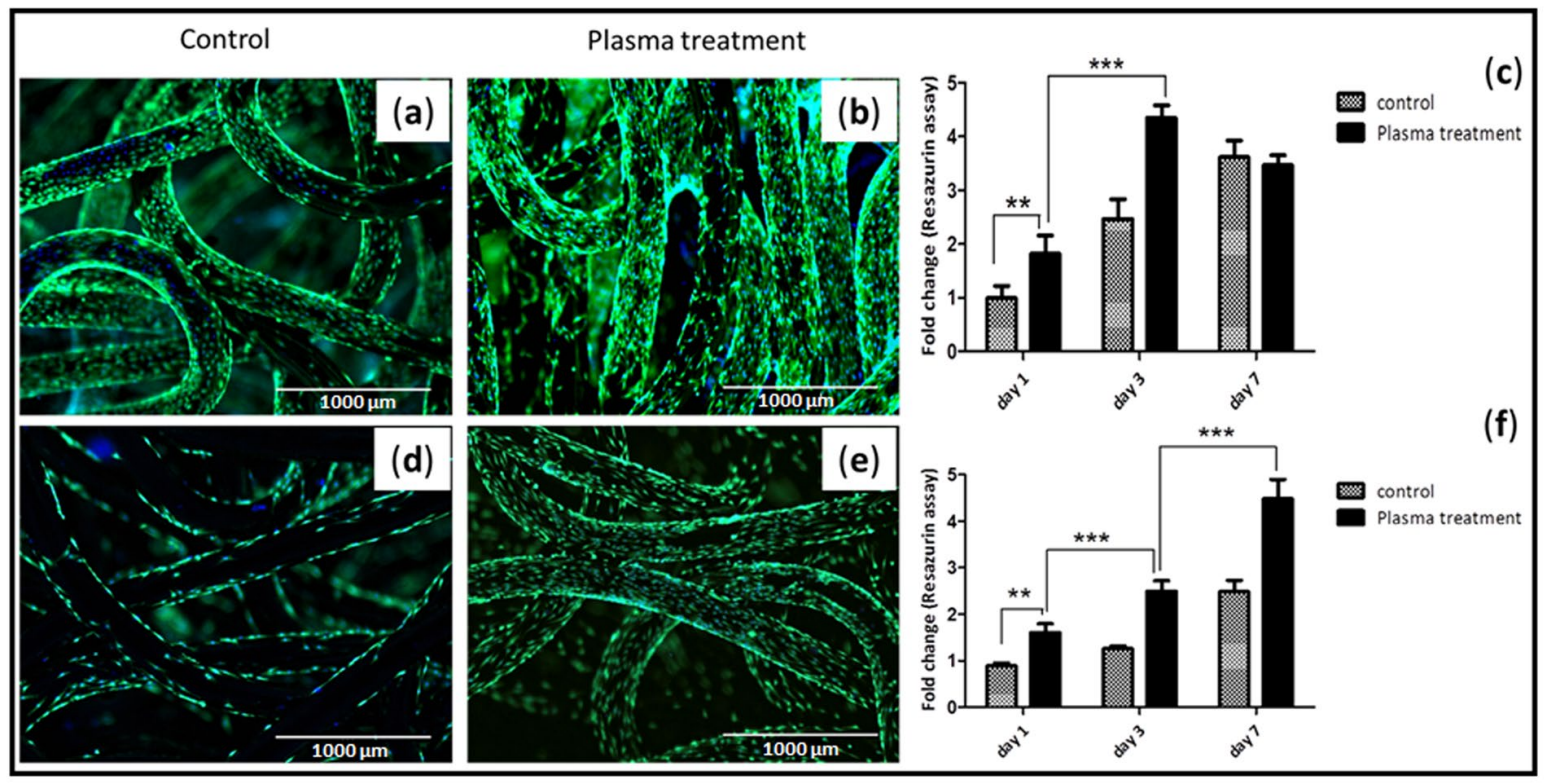
Effect of plasma treatment on cell viability and proliferation. Live dead staining - viable cells; calcein-AM (green) and nucleus; Hoechst 33342 (blue) staining of SCP1 cell and human primary chondrocytes upon seeded on the (**a**,**d**) non-plasma treated scaffold; (**b**,**e**) oxygen plasma treated scaffold respectively. Scale bar = 1000 µm. More cells are evident on the oxygen plasma treated scaffolds compare to the non-treated scaffolds; (**c**,**f**) Resazurin conversion – indirect assay for cell proliferation of SCP1 cells and human primary chondrocytes respectively. Fold increase was significant upon oxygen plasma treatment. Data represented as mean ± SEM; analyzed by Mann Whitney test. P ≤ 0.05 was considered to be minimum level of significance. *p ≤ 0.05, **p ≤ 0.01, ***p ≤ 0.001.

**Figure 8:**
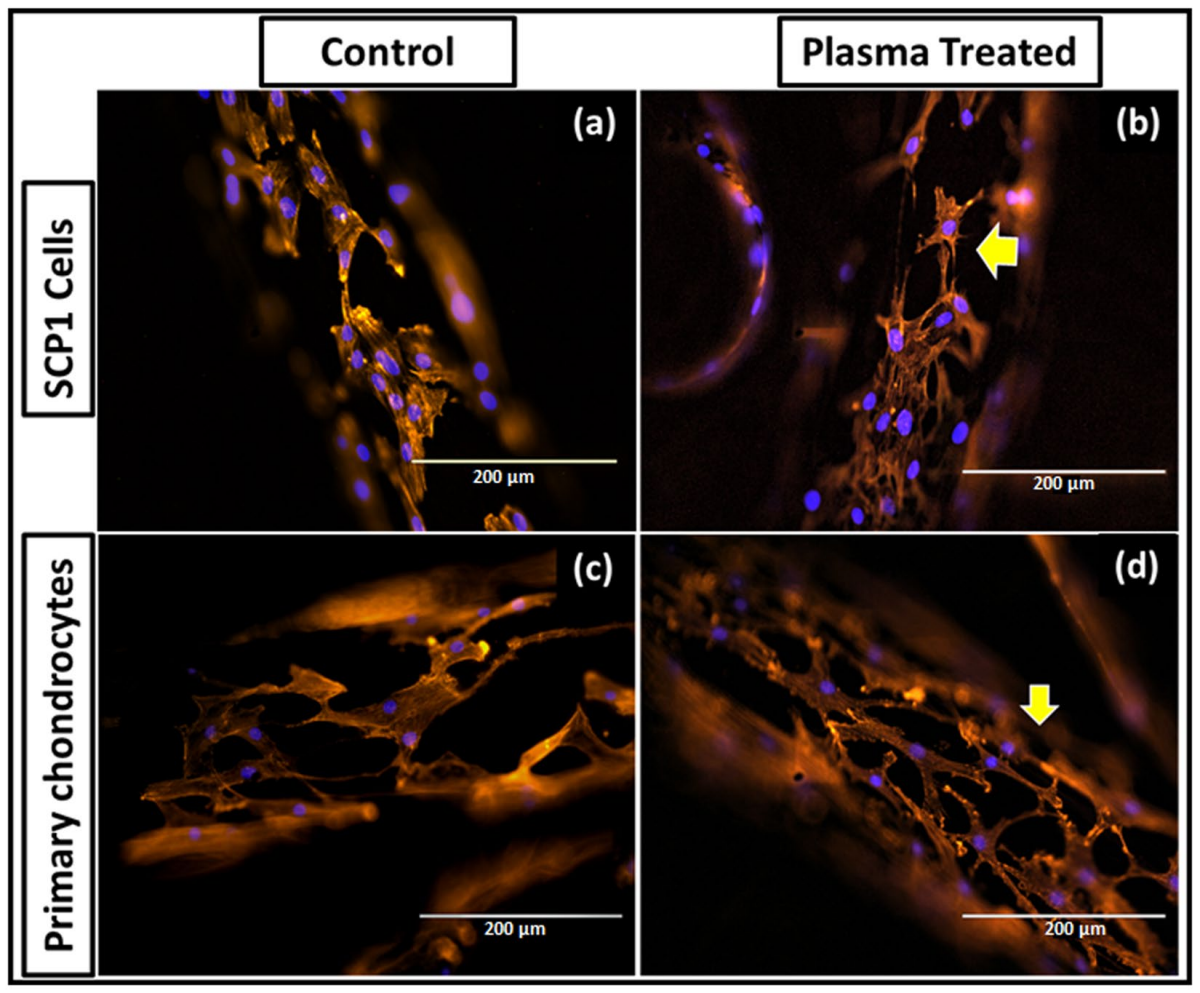
Effect of plasma treatment on cell spreading. Actin-Phalloidin; alexa 555 (red) and nucleus; Hoechst 33342 (blue) fluorescent stained SCP1 cells day 3 post seeded on the non-plasma treated (**a**), oxygen plasma treated scaffold (**b**); human primary chondrocytes seeded on the non-plasma treated (**c**), oxygen plasma treated scaffold (**d**). Scale bar = 200 µm.

To elucidate the effect of scaffold pretreatment with oxygen plasma on the cellular response, cell viability and proliferation were assessed post treatment. Figure 7 represents viable cells at day 3; calcein-AM (green fluorescence) and nucleus (blue fluorescence) staining as indicated in plasma treatment and control group, where more cells were evident on plasma treated scaffolds compare to non-treated scaffolds. There was no substantial change in terms of cell adhesion, however cell growth was found to be enhanced upon plasma treatment (Fig. 7c,f). Significant fold increase was observed in the fluorescence intensity and subsequent cell number at day 3 and 7 when it normalized with those on day 1. Additionally, significant difference between the two groups was observed (Fig. 7c,f). SCP1 cell proliferation at day 7 was almost similar in both controls and plasma treated scaffolds, whereas primary chondrocyte cell growth was higher upon plasma treatment; showing a positive effect of oxygen based plasma treatment. Furthermore, we confirmed that the plasma treatment had an influence on the cell morphology as the cells appeared more spread out with longer extensions following actin (red fluorescence) and nuclear staining (blue fluorescence) as observed on plasma treated scaffolds (Fig. 8).

## Discussion

Titanium and its alloys are well established in the biomedical field due to their worthy mechanical properties, biocompatibility and low immunogenicity^6^. To further pursue this goal, we used titanium scaffold with a unique knitted geometry simulating the porous structure, precisely to create suitable conditions for cell attachment and tissue ingrowth. Ideally the scaffold should retain their dimensions, integrity and strength along with the biological integrity during the process of wound healing/tissue regeneration after the implantation^10^. One of the critical reasons for implant failure is the mobility of the implant leading to local abrasion and immune reaction^18–20^. Therefore the manufacturing procedure and surface modifications have a definite impact on the quality, performance and integrity of the implant *in vivo* ^12,21^. In a previous study, Kettler *et al*. showed that cyclic loading of this knitted titanium scaffold did not cause implant expulsion. A potential advantage of using porous titanium knitted scaffold was its elasticity that in the range of that of cancellous bone. This upon mismatch possibly leads to stress shielding around the scaffold causing bone atrophy and induction of local inflammation which ultimately results in implant failure. Nevertheless, with respect to biomechanics, one of the most vital factors to be considered is wear and corrosion resistance. Wear debris particles have been reported to have role to induce toxicity, especially particle mediated inflammation triggers the granuloma cascade, necrosis and/or fibrosis all together causing the aseptic loosening^18,22^ and therefore the excessive amount of the wear debris particles within the host system can cause adverse cellular host reactions. The intensity of local inflammation mainly depends on debris characteristics such as: particle load (size and volume) and chemical reactivity. Wear debris prone implant failure is prime suspect in the field of arthroplasty as attrition evoking immune reaction cannot be omitted^19^. In our study, the purpose of explicitly considering the electro-polished knitted scaffold was to emphasize the fact of wear resistant material goods. Moreover, the purpose of the wear debris analysis was not the elucidation of such body reactions, but rather to obtain a better understanding of the morphology of titanium wear particles. This information may have future implications for the design and adapting of the scaffold employed in musculoskeletal implants. Consequently, electro-polishing resulted in a substantial reduction of overall abrasion production which can ensure prolonged functional longevity of the scaffold *in vivo*.

Additionally, the success of an implant also depends on its biocompatibility which maintains its integration with the surrounding tissue and therefore will define its functionality *in vivo* ^21^. In order to mimic the appropriate conditions, the human bone marrow derived mesenchymal stem cells and human primary chondrocytes were used for all experiments. As an early cell response towards the implant site, cell adhesion and cell proliferation were studied considering the scaffold surface characteristics *i.e*. surface roughness and wettability, as the desired physical properties of the scaffold are vital for its effective incorporation on the system. Owen *et al*. has described in earlier report that it is possible to modulate specific cell behavior by changing the surface topography^23^. Previously it has been reported that the optimum pore size range of 200–500 µm is suitable for tissue ingrowth^24^. However, there is no precise specification for optimal pore size that has been proved. He *et al*. earlier reported that the wire diameter influences the pore size^25^. Based on these reports, the scaffold used in our study presumed to have a favorable pore size distribution which can promote tissue in growth. Since it is possible that cell behavior could vary with pore size variation, in our study, we maintained uniform defined geometry of the porous knitted scaffold with a pore size of 150–400 μm and porosity roughly about 67.67 ± 0.824%, aiming to evaluate the scaffold cytocompatibility.

In order to obtain a realistic biomechanics prediction of *in vivo* situation, stress distribution within tissue–implant interface and its physio-chemical characteristics needs to be further analyzed. Scaffold surface properties such as surface roughness, composition, topography are known to influence the nature of cell adhesion which thereby directs the process of cell proliferation and differentiation. The role of surface roughness in deciding cell behavior is still debatable. A study by Hallab *et al*. shows that the surface roughness and the surface energy play crucial roles during initial cell response at the cell-material interface. Also, changes in the surface topography and increase in the roughness decreases the cell proliferation^26^. Surface energy of the material on contrary is proved to be a prime factor than the surface roughness in regulating the cell adhesion on the material surface^27^. Moreover in our study, the decrease in surface roughness upon electro-polishing treatment did not play any major role in initial cell attachment and neither affected the cell growth rate. Based on the obtained results, change in the scaffold surface roughness as observed in the study seems to have negligible effect on the cell proliferation which supports the possibility that the surface energy is a dominating factor in promoting cell growth^27^. Nonetheless, surface roughness is highly required from a biomechanics prospective for a long term function of load bearing implant as it limits the micro crack formation and leads to better load distribution^28^. The host reaction towards the scaffold is mainly dictated by the adsorbed proteins during initial contact with body fluids. Since this reaction is preferential, it is influenced by the wide range of scaffold physical properties like surface roughness, topography, creating a porous material with high interconnectivity, wettability etc. For example, smooth and double acid-etched micro rough TiAl6V4 surfaces showed contact angles of 62° and 57°, respectively^16^. Moreover, surface modifications aimed at improving its bioactivity can be achieved by modification of these physical parameters. Therefore, desire scaffold structure and surface modifications are advantageous with respect to the site of scaffold in order to contribute the clinical success rate.

From clinical aspect, this study was aimed at investigating the biocompatibility of the proposed knitted titanium scaffold. Concerning the load bearing applications, targeted implant prerequisites to get stabilized first for which cell reaction towards the material surface is an important factor of selection. From the economic trait, the sooner tissue integration the better is the healing process. However, it is yet to be clear from clinical aspect that what all parameters are prime objects for faster healing. Therefore, it is important to develop scaffolds with appropriate surface properties. Here it could be proposed that, surface modifications should be mainly based on the site of implant within the body *in vivo*. In musculoskeletal prosthetics, a hydrophilic surface favors the tissue integration^29^.

Surface wettability is another important factor that affects the extent of cellular responses on the implant surface. Hydrophilization and sterilization of biomaterial surfaces due to radio-frequency glow discharge (RFGD) plasma treatment is well known phenomena and occur due to cleaning of contaminants and by achieving of a high energy state^30^. The foremost intention of the surface modification by plasma treatment in our study was to improve the scaffold wettability and ultimately modulate cell attachment and proliferation of SCP1 cells and primary chondrocytes. Importantly, plasma treatment showed a positive influence on cell behavior in terms of cell viability, proliferation and the morphology; thus indicating a crucial role in mediating the cell response towards the scaffold surface. Further, studies on cell differentiation could be supportive for further understanding of the observed cell behavior with respect to SCP1 cells.

In summary, we have explored the potential of knitted titanium to allow viability and proliferation of adherent-cells. It has further shown that cells response with respect to viability and proliferation indeed remain unaffected upon electro-polishing treatment. This suggests that it is the wettability and not the surface roughness which could be the main cause for modulating the response at cell material interaction. Using oxygen plasma treatment, we have further demonstrated that the proliferation of the adherent-cells on the scaffold has been elevated as compared to non-treated conditions. Though our results shows *in vitro* cytocompatibility potential of knitted titanium scaffold, it needs to be verified further through *in vivo* studies to project it as a potential treatment option in the clinical settings.

## Conclusion

This *in vitro* study demonstrated the potential of the knitted titanium scaffold as a possible replacement of intervertebral nucleus pulposus, while revealing some of the weaknesses especially because of the lack *in vivo* outcomes. Nonetheless, these results pave the way for establishing knitted titanium scaffold as an alternative solution for musculoskeletal tissue engineering, involving cell-material interaction. Combination of oxygen plasma treatment considerably affects cell proliferation compared to non-hydrophilized electro-polished scaffolds alone and thus seems to be a suitable additional surface modification for better tissue integration. It can be concluded that knitted titanium wire scaffold has shown a promising potential intervertebral nucleus substitute and can be used efficiently with oxygen plasma treatment to improve the tissue integration process.

## Materials/Methods

### Knitted titanium scaffold and surface modification

Knitted titanium scaffold was prepared as previously reported^31^. Briefly, medical grade titanium alloy (Ti6Al4V) wires with diameter of 0.25 mm were knitted to produce a mesh like cuboidal disk structure with 5 mm height and 14 × 11 mm dimension (Buck Co & GmbH, Bondorf, Germany), volumetric porosity of 67.67 ± 0.824%, and density of 0.75 mg/cm^3^ and used in this study. Electro-polishing of the scaffolds was used to generate smooth wear resistant surface with defined finish. Scaffolds were cleaned with 1% Triton-X followed by ultrasonication in reagent grade acetone, iso-propanol, ethanol and distilled water^32^ respectively for 15 min to remove impurities generated either during manufacture or due to electro-polishing. The scaffolds were then dried at room temperature, followed by sterilization at 121 °C and 15 PSI for 20 min before use.

Electro-polished scaffolds were further processed by oxygen plasma treatment, to yield hydrophillized surfaces. Single wires as well as scaffolds were treated in a low pressure Dentaplas PC plasma system (Diener electronic, Ebhausen, Germany). After generation of 0.3 mbar vacuum within 2 min, 160 W plasma was ignited at an oxygen flow of 3 sccm and applied for 15 min at 40 °C.

### Scanning Electron Microscopy (SEM)

Surface morphology of the knitted scaffold was determined using SEM (JEOL, JSM-7100F, Japan) after gold sputter coating. Images were recorded at an accelerating voltage of 15 kV. The surface roughness of unpolished and electro-polished scaffold was evaluated at 15,000× magnification. Chemical analysis of the material was studied by energy dispersive X-ray spectroscopy (EDX). Survey spectra were collected at pass energy of 80 eV.

All the SEM pictures were then used further to determine the pore size. The Image J software was used (https://imagej.nih.gov/ij/). The software was calibrated using the micron scale bar of each picture. An average pore size was determined by measuring the diameter of 50 different pores. Pores were identified as areas of void space bounded by fibers on all sides at or near the same depth of field, while their long and short diagonal axes were measured together to serve as their pore size range.

### Optical profilometry

The effect of electro-polishing treatment on the scaffold surface roughness was analyzed using non-contact profilometry (Contour GT-K, Bruker, USA). Breifly, electro-polished and unpolished scaffold surfaces were scanned using 20× objective, area of 240 µm × 180 µm, and calculating the average roughness (Ra) of the surface using Vision-64 (Bruker) surface analysis software.

### Sessile Drop Contact Angle Measurement

The contact angle measurements of unpolished and electro-polished scaffold were performed using surface goniometer (OCA 15 plus, Data-Physics Instrument GmbH, Germany) equipped with automatic drop application and contact angle evaluation.

Wettability of non-treated and plasma-treated electro-polished scaffolds was analyzed by automatic drop application and contact angle evaluation by a drop shape analysis system (DSA 10-Mk2, Kruess, Germany). Water droplets (2 µl) were deposited on the surfaces and the drop shape and infiltration behavior was recorded by a video camera (1 frame/s). If not infiltrated, contact angles of the sessile drops were measured 20 seconds after the initial surface contact analyzed from drop shapes by applying the circular segment method (circle fitting) implemented in the Kruess software (Kruess, Germany).

### Dynamic Contact Angle Measurement

Dynamic wetting of the plasma-treated and untreated electro-polished single wires was investigated tensiometrically (KSV Instruments Ltd., Finland) using Wilhelmy balance method^15^. Prior to measurement in water, its surface tension was determined with a standardized, fully wettable platinum plate (39.4 mm wetted length; 19.6 mm width; 0.1 mm thickness). The samples were immersed and emersed with speed of 10 mm/min for one complete cycle with an immersion depth of 10 mm. From the recorded force loops, dynamic advancing (θadv) and receding (θrec) contact angles were calculated by extrapolating the corresponding immersion and emersion force/length-branches of the hysteresis loops to the zero immersion depth according to the following equation:

1 $$F=\gamma \,L\,\cos \,\theta $$

where γ: surface tension of the liquid, L: perimeter (wetted length) of the wires, and F: force at zero immersion depth where buoyancy is not apparent.

### Compression analysis

The compression of scaffolds with 5 mm height and 14 × 11 mm dimension were analyzed^33^ using uniaxial compression test by a mechanical tester (BiSS Ltd, India). The scaffold was placed between the two arms of load frame and compressed until mechanical failure with a speed of 0.5 mm/min. The applied force and displacement were continuously recorded during testing. The Young’s modulus was calculated using the formula:

2 $${\rm{E}}=({\rm{F}}{\rm{/}}{\rm{A}}){\rm{/}}({\rm{\Delta }}{\rm{l}}{\rm{/}}{\rm{l}})\,{\rm{kPa}}$$

where E: Young’s modulus, F: applied force, A: cross-sectional area of the scaffold, l: initial length of the test sample, and Δl: change in length under the compressive force.

### Porosity

The porosity of the knitted titanium scaffold was directly obtained by mass–volume calculation method^34^ using following equation:

3 $${\rm{P}}=1{\rm{-}}{\rm{M}}{\rm{/}}({\rm{V}}{\rm{\times }}\rho {\rm{s}})\times {\rm{100}}$$

where P: porosity; M: weight of the scaffold; V: volume of the scaffold; ρs: density of the scaffold.

### Wear debris particle analysis

The wear test of the scaffold was done by Buck co & GmbH. During the test particles were collected in suitable lubricant, which then analysed further. Wear debris particle morphology and size was studied using SEM (Zeiss DSM962, Germany). Briefly, samples were prepared by precipitating the particles upon centrifugation at 10,000 rpm for 40 min (to remove the traces of lubricant) following resuspension in 70% ethanol. Spin coating was performed to deposit a uniform thin particle layer on a piece of a Si wafer (70 rps; t = 20 s; V = 100 µl, K.L.M. Spin Coater, Germany). To avoid agglomeration, the suspensions were sonicated for 10 min prior to spin coating process. Upon solvent evaporation following coating, the samples were sputter coated with gold.

### Cell culture

Human immortalized bone marrow derived mesenchymal stem cell line (SCP1 cells)^35^ was cultured in α-MEM (minimal essential medium; GIBCO, Germany) containing 1 g/L glucose supplemented with 10% FBS (fetal bovine serum; GIBCO, Germany) and 1% penicillin/streptomycin (Sigma, Germany) 10,000 units/ml 10 mg/ml respectively (P/S) in a humidified incubator (37 °C and 5% CO_2_).

Human primary chondrocytes were isolated^36^ from the donor samples (BG Klinik, Tübingen) after complete ethical clearance (338/2015BO2). Briefly, cartilage tissue was removed from the femoral condyles and transferred to Dulbecco’s modified essential medium (DMEM)/F12 (1:1) medium containing 25 µg/ml Fungizone®Antimycotic and 1% P/S, Phosphitan: analogue of ascorbic acid (25 mg/mL; Sigma, Germany). The tissue was cut into small pieces, washed with phosphate-buffered saline (DPBS) and digested with collagenase II (GIBCO, Darmstadt, Germany) 1500 U/ml in chondrocyte medium for 16–24 hr at 37 °C with agitation (100–120 rpm). The suspension was passed through 70 μm cell strainer, centrifuged, resuspended in chondrocyte medium and incubated at 37 °C with 5% CO_2_. At the time of seeding, sterile scaffolds were placed into each well of 24-well plates, pre-conditioned with media for 30 min; after which medium was aspirated. Cells were trypsinized (Trypsin-EDTA; 0.05%/0.02%, Sigma, Germany), centrifuged and resuspended to achieve a seeding density of 2 × 10^5^ cells per scaffold. Cells seeded scaffold kept in incubator initially for 3 hr to allow cell attachment and thereafter, 700 µl of additional medium was added to each well and incubated further.

### Resazurin assay

Cell proliferation was calculated using resazurin conversion assay^37^. Briefly, cells seeded scaffolds were incubated with a 1:10 volume of sterile resazurin (Sigma, Germany) working solution (0.025% in DPBS) at 37 °C for 30 min. Resulting fluorescence (ex/em = 540/590 nm) was then measured spectroscopically (BMG, Offenburg, Germany) and corrected to background control. Fluorescence value directly correlates with a cell number. Standard curve was generated which was then used to calculate the cell proliferation.

### Live-dead staining

Cell viability was determined at day 3 post seeded by intracellular esterase activity using calcein-AM and ethidium homodimer to determine dead cells^14^. Briefly, cells were stained with 2 µM calcein-AM (Sigma, Germany), 4 µM ethidium homodimer (Sigma, Germany) and Hoechst 33342 (1:1000 in DPBS, Sigma, Germany) at room temperature (RT) for 30 min following DPBS wash. Images were taken using epifluorescence microscopy (Life technologies, Darmstadt, Germany).

### Cell Morphology

Cells seeded scaffolds were washed with DPBS and then cells were fixed using 4% paraformaldehyde for 10 min at RT after which cell permeabilisation with 0.25% Triton-X 100 solution (Sigma, Germany) for 20 min at RT. 1% BSA (Bovine Serum Albumin, Sigma, Germany) blocking solution was added and incubated at RT for 1 hr. Further, it stained with Alexa Fluor® 555 Phalloidin (1:500, Santa Cruz, Germany) and Hoechst 33342 (1:1000) for actin fibers and cell nuclei respectively and incubated in dark for 45 min. Images were captured using epifluorescence microscopy.

### Statistical analysis

Analysis was done using GraphPad Prism 5.0 software (El Camino Real, CA, USA) and PAST.exe (http://folk.uio.no/ohammer/past/index). Statistical significance of all experiments that were done in triplicates was analyzed using the Kruskal-Wallis H-test followed by a Dunn’s test and Mann Whitney test. Minimum level of significance: P ≤ 0.05. *p ≤ 0.05, **p ≤ 0.01, ***p ≤ 0.001.
